# *TM6SF2* and *MBOAT7* Gene Variants in Liver Fibrosis and Cirrhosis

**DOI:** 10.3390/ijms20061277

**Published:** 2019-03-14

**Authors:** Viktorija Basyte-Bacevice, Jurgita Skieceviciene, Irena Valantiene, Jolanta Sumskiene, Vitalija Petrenkiene, Jurate Kondrackiene, Dalius Petrauskas, Frank Lammert, Juozas Kupcinskas

**Affiliations:** 1 Department of Gastroenterology, Lithuanian University of Health Sciences, LT-50009 Kaunas, Lithuania; vbacevice@gmail.com (V.B.-B.); irena.valantiene@fc.lsmuni.lt (I.V.); jolanta.sumskiene@fc.lsmuni.lt (J.S.); vitalija.petrenkiene@lsmuni.lt (V.P.); jurate.kondrackiene@fc.lsmuni.lt (J.K.); dalius.petrauskas@fc.lsmuni.lt (D.P.); 2 Institute for Digestive Research, Lithuanian University of Health Sciences, LT-50009 Kaunas, Lithuania; jurgita.skieceviciene@lsmuni.lt; 3 Department of Medicine II, Saarland University Medical Center, Saarland University, 66421 Homburg, Germany; frank.lammert@uks.eu

**Keywords:** hepatic fibrosis, liver cirrhosis, *MBOAT7*, *TM6SF2*, *PNPLA3*, gene polymorphism

## Abstract

Previous large-scale genetic studies identified single nucleotide polymorphisms (SNPs) of the *TM6SF2* and *MBOAT7* genes as risk factors for alcoholic liver cirrhosis and non-alcoholic fatty liver disease. In this study, we tried to evaluate the association between *TM6SF2* variant *rs58542926* and *MBOAT7* variant *rs641738* and the risk of hepatic fibrosis or liver cirrhosis of different etiology. In parallel, we also aimed to evaluate whether these two SNPs modify the effects of the *PNPLA3 rs738409* risk variant for the development of hepatic fibrosis and liver cirrhosis. The study was conducted at the Department of Gastroenterology, Lithuanian University of Health Sciences Hospital, and included 334 patients with liver cirrhosis, 128 patients with liver fibrosis, and 550 controls. SNPs were genotyped by quantitative PCR, using TaqMan allelic discrimination assays. Overall, *TM6SF2 rs58542926* as well as *MBOAT7* *rs641738* were not linked to hepatic fibrosis, alcohol or hepatitis C virus induced liver cirrhosis in an Eastern European population. These genetic variations also did not mediate the effect of *PNPLA3 rs738409* SNP for liver developing liver fibrosis or liver cirrhosis.

## 1. Introduction

Liver cirrhosis is one of the dominant causes of global disease burden and is associated with life threatening complications [1]. In 2016, liver cirrhosis accounted for almost 39 million disability adjusted life years (DALYs), or 1.6% of global DALYs, and resulted in more than three million deaths worldwide [2,3]. The most common causes of chronic liver injury are hepatitis C virus (HCV) and hepatitis B virus (HBV) infections, alcohol misuse, non-alcoholic steatohepatitis, autoimmune hepatitis and other conditions [4,5,6]. To date, individual variability for susceptibility and severity of liver injury is still not completely understood. An increasing burden of liver diseases, high individual variations in disease course and rapidly developing genotyping technologies lead to multiple genetic studies that showed the importance of genetic factors in the development and progression of chronic liver injury [7,8,9].

Genome wide association studies (GWAS) are now widely used to identify associations between single nucleotide polymorphisms (SNPs) and the risk of different diseases employing next-generation sequencing based techniques. Over the last decade a significant number of GWAS have been performed in the field of hepatology [10], revealing the genetic predisposition for primary biliary cholangitis, primary sclerosing cholangitis, steatohepatitis and other liver diseases [7]. Several groups including ours have previously shown that the patatin-like phospholipase domain containing 3 (*PNPLA3*) gene SNP is linked with liver injury [11,12]. More recently, transmembrane 6 superfamily member 2 (*TM6SF2*) and membrane-bound O-acyltransferase domain-containing protein 7 (*MBOAT7*) gene polymorphisms have been identified as risk factors for alcoholic liver cirrhosis [12]. *TM6SF2* variant *rs58542926* has also been associated with an increased risk of developing advanced hepatic fibrosis and cirrhosis in patients with non-alcoholic fatty liver disease (NAFLD) [13]. Furthermore, the *MBOAT7* polymorphism *rs641738* was associated with the severity of liver fibrosis in individuals with chronic hepatitis C virus infection [14] and NAFLD [15]. In a recent study, *MBOAT7 rs641738* SNP was also reported as an independent predictor of severe liver steatosis in patients with chronic hepatitis C [16].

In this study we analyzed the association between *TM6SF2* variant *rs58542926* and *MBOAT7* variant *rs641738* and the risk of hepatic fibrosis or liver cirrhosis of different etiology in an Eastern European patient cohort. Additionally, we also aimed to evaluate whether these two SNPs modify the effects of the *PNPLA3 rs738409* risk variant on the development of hepatic fibrosis and liver cirrhosis.

## 2. Results

### 2.1. Characteristics of Study Participants

Table 1 presents the demographic and clinical characteristics of the study group. The mean age of patients with liver cirrhosis was 51.8 years, and they were significantly older (*p* < 0.001) than patients with liver fibrosis and controls. Men were predominant in the liver fibrosis group (61.7%. *p* < 0.05). The major cause of liver cirrhosis was alcohol, the second most common cause was chronic HCV infection. In the liver fibrosis group, the most common cause of liver injury was HCV infection. To eliminate the potential bias of differences in age and sex distribution among the groups, these parameters were included as covariates in logistic regression analysis. The frequencies of alleles and genotypes were in Hardy–Weinberg equilibrium for all SNPs analyzed (all *p* > 0.05).

### 2.2. TM6SF2 rs58542926 and MBOAT7 rs641738 SNPs

Allele and genotype distributions for the *TMS6SF2 rs58542926* and *MBOAT7 rs641738* polymorphisms in patients with different etiology of liver cirrhosis, hepatic fibrosis and the control groups are presented in Table 2 and Table 3. *TM6SF2 rs58542926* allele frequencies in controls, patients with hepatic fibrosis, liver cirrhosis, alcohol-induced and HCV-induced cirrhosis were: T allele 7.5%, 5.5%, 8.4%, 8.1% and 7.5%; C allele 92.6%, 94.5%, 91.6%, 91.8% and 92.5%, respectively. None of the *TM6SF2 rs58542926* alleles or genotypes were linked with the risk of developing liver fibrosis or cirrhosis. *MBOAT7 rs641738* SNP alleles and genotypes showed similar distributions across all four groups of the study. In controls, the frequencies of T and C alleles were 43.3% and 56.7%, in the liver fibrosis group 41.4% and 58.6%, in the cirrhosis group 44.6% and 55.4%, in alcohol-induced cirrhosis 42.1% and 57.9%, in HCV-induced cirrhosis 45.8% and 54.2%, and in patients with cirrhosis due to other causes 51.2% and 48.8%, respectively. No significant differences between *MBOAT7 rs641738* alleles and genotypes among the different study groups were observed.

### 2.3. Combined Analysis of PNPLA3 rs738409 and MBOAT7 or TM6SF2 SNP Genotypes

In this part of the study we used genotyping data from our previous study on *PNPLA3 rs738409* and liver fibrosis/cirrhosis [11]. Table 4 and Table 5 present the data for the combined *PNPLA3 rs738409* and *MBOAT7* or *TM6SF2* genetic variants. This analysis showed no significant differences between combined *MBOAT7* and *TM6SF2* SNPs and *PNPLA3* risk genotypes.

## 3. Discussion

In the current study, we aimed to evaluate the associations between *TM6SF2 rs58542926* and *MBOAT7 rs641738* genes polymorphisms and the risk of hepatic fibrosis and liver cirrhosis. Overall, *TM6SF2* and *MBOAT7* SNPs were not linked with hepatic fibrosis and liver cirrhosis of different etiology within our study. Additionally, we ran combined analysis to assess whether *TM6SF2* and *MBOAT7* SNPs mediate the risk of liver fibrosis or liver cirrhosis in the presence of certain *PNPLA3* genotypes. The effects of *PNPLA3* have been well established in previous research, but we wanted to see if the risk of liver fibrosis is changed by a certain combination of *PNPLA3* and *TM6SF2* or *MBOAT7* genotypes. To the best of our knowledge, this is the first study of an Eastern European population that assessed the impact of *TM6SF2* rs58542926 and *MBOAT7 rs641738* on developing liver injury.

*TM6SF2* gene is important for the secretion of very-low density lipoprotein from hepatocytes. Its reduced function leads to increased liver triglyceride and lipid droplet content which contributes to NAFLD [17,18,19]. Genetic variations within *TM6SF2* gene have been linked with a number of liver conditions; however, reported results are varying in between. *TM6SF2 rs58542926* was identified as a modifier of hepatic fibrogenesis [13] and was associated with histological severity of steatosis, increased hepatic inflammation and fibrosis [20,21]. Coppola et al. demonstrated that *TM6SF2 rs58542926* is linked with development of severe liver steatosis in patients with chronic hepatitis C (CHC), although no significant association between *TM6SF2* and severe liver necro-inflammation or fibrosis was found [16]. Further, in a later larger study, Milano et al. concluded that *TM6SF2 rs58542926* not only had an impact on liver steatosis, but was also relevant in the development of severe fibrosis in individuals with CHC [22]. Meanwhile, Sookoian et al. showed that *rs58542926* has a modest effect on NAFLD [23]. Moreover, *TM6SF2* genotype did not impact histological features with biopsy-proven NAFLD in Japanese patients [24]. To confirm the effect of *TM6SF2* on fibrosis in NAFLD, CHC and CHB patients, Eslam et al. used a large cohort of Caucasians and found that *rs58542926* has more influence on the serum metabolic profile and predisposed hepatic steatosis, rather than acting directly on liver inflammation and fibrosis [25]. Another large cohort with genotype 1 HCV or with NAFLD found no association between the *TM6SF2* variant and hepatic fibrosis [26]. The results of our study showed that direction for *TM6SF2 rs58542926* between fibrosis and cirrhosis groups was different, but the significance level was not reached and this genetic variant alone or combined with *PNPLA3* risk genotype was not linked with liver fibrosis or cirrhosis.

The association of *MBOAT7 rs641738* SNP with the entire spectrum of NAFLD in individuals of European descent was first reported by Mancina et al. [27]. The same *MBOAT7* genetic variant was not associated with hepatic steatosis, but significantly linked to hepatic fibrosis by Krawczyk M. and colleagues [15]. In an Asian population, *MBOAT7 rs641738* was not linked with risk of developing NAFLD [21]. Yet another study found no association between *rs641738* and liver steatosis, but an association with hepatic inflammation and fibrosis in CHC was detected [14]. Nevertheless, no recent studies were conducted to evaluate the significance of this genotype on hepatic fibrosis or cirrhosis of different etiology. Varying findings of different studies clearly show that the final outcome of chronic liver injury may have a different effect from genetic factors in different populations. Within our study *MBOAT7* locus *rs641738* was not linked with liver fibrosis or cirrhosis. When combining different genotypes of this SNP with a well know *PNPLA3 rs738409* variant no significant differences were observed.

There are certain limitations associated with the design of our study that need to be acknowledged. The major limitation of our study is related to etiological heterogeneity of liver cirrhosis cohort and not a large number of patients within the subgroup analysis. Most of our patients in the liver fibrosis group had HCV induced fibrosis. Moreover, relatively small sample sizes within combined *PNPLA3* SNP sub-analysis needs further re-evaluation in larger well-defined cohorts in order to evaluate observed synergistic effects of different genetic variations.

## 4. Materials and Methods

### 4.1. Patients

The *TM6SF2 rs58542926* and *MBOAT7 rs641738* genotyping study included 1012 individuals (550 controls, 334 patients with liver cirrhosis and 128 patients with liver fibrosis). For combined analyses of carriers of *PNPLA3 rs738409* risk genotype, we used data from our previous study with the same cohort [11] and included 798 individuals (401 controls and 269 patients with liver cirrhosis and 128 patients with liver fibrosis). Patients with liver cirrhosis and hepatic fibrosis were recruited during the period 2012–2017. Diagnosis of liver cirrhosis was established by standard clinical features, laboratory tests, and radiological imaging. Hepatic fibrosis was diagnosed based on histological evaluation of liver biopsy specimens. Liver fibrosis group consisted of the patients that had stage I to stage III fibrosis in histological evaluation according to the METAVIR score [28]. All individuals that had fibrosis stage IV according to Metavir score were transferred to liver cirrhosis group. Control samples came from our previous genotyping study on the prevalence of HFE mutations in the Lithuanian population and included 550 voluntary, unrelated Lithuanian blood donors [29]. The genotyping was conducted at the Institute for Digestive Research at Lithuanian University of Health Sciences Hospital. The study was carried out in line with the 1975 Declaration of Helsinki (6th revision, 2008). All patients and controls have written an informed consent to participate in this study. The study was approved by Regional Kaunas Ethics Committee (Protocol No. BE-10-2, approval date: 08 March 2011).

### 4.2. Genotyping

Genomic DNA from samples was isolated from whole blood mononuclear cells using a salting-out method and stored at −20 °C until analysis, as described previously [30]. *TM6SF2 rs58542926* and *MBOAT7 rs641738* SNPs were genotyped by real-time PCR (RT-PCR), using TaqMan^®^ allelic discrimination assays with a 7500TM Fast real-time PCR system (Life Technologies, Carlsbad, CA, USA).

### 4.3. Statistical Analysis

*TM6SF2* and *MBOAT7* alleles and genotypes frequencies between cases and controls were compared by Pearson’s goodness-of-fit χ^2^ and Fisher exact tests. Associations between control and cases groups with SNP alleles and genotypes were calculated using logistic regression analysis with adjustment for age and sex. Table 2 summarizes the adjusted odds ratios (aORs) and 95% confidence intervals (CI). Age between groups was compared using analysis of variance (ANOVA). Gender distributions were compared using the χ^2^ tests. For additional analysis, we divided the cohort in two groups with respect to *PNPLA3 rs738409* genotypes. *p*-value was adjusted for multiple comparisons and the value of <0.025 (0.05/2) was considered to be statistically significant. Statistical analysis of the genotyping data was performed using PLINK software version 1.07 [31].

## 5. Conclusions

In conclusion, *TM6SF2 rs58542926* as well as *MBOAT7 rs641738* were not linked to hepatic fibrosis, alcohol or hepatitis C virus induced liver cirrhosis in an Eastern European population. These genetic variations also did not mediate the effect of *PNPLA3 rs738409* SNP on the development of liver fibrosis or liver cirrhosis.

## Figures and Tables

**Table 1:** Characteristics of patients with liver cirrhosis, hepatic fibrosis and controls.

	Liver Cirrhosis (*n* = 334)	Liver Fibrosis (*n* = 128)	Controls (*n* = 550)	*p* Value
Age (mean ± SD), years	51.8 ± 13.2	47.2 ± 13.4	47.3 ± 9.0	<0.001
Gender, *n* (%)				
Male	166 (48.3%)	79 (61.7%)	271 (49.3%)	<0.05
Female	168 (51.7%)	49 (38.3%)	279 (50.7%)	
Aetiology of liver disease, *n* (%)				
Alcohol	171 (51.2%)	8 (6.25%)		
HCV	120 (35.9%)	112 (87.5%)		
Other causes	43 (12.9%)	8 (6.25%)		
HBV	17 (5.1%)	5 (3.9%)		
HCC	13 (3.9%)			
Autoimmune	8 (3.9%)	3 (2.3%)		
Hemochromatosis	4 (1.2%)			
Wilson’s disease	1 (0.3%)			

HBV—hepatitis B virus, HCV—hepatitis C virus, HCC—hepatocellular carcinoma.

**Table 2:** Distribution of the *TMS6SF2* and *MBOAT7* SNPs alleles and genotypes in liver fibrosis and cirrhosis groups.

Allele/Genotype	Controls (*n* = 550)	Fibrosis (*n* = 128)		Cirrhosis (*n* = 334)			
	*n* (%)	*n* (%)	aOR (95% CI)	*p*	*n* (%)	aOR (95% CI)	*p*
*rs58542926* (*TM6SF2*)							
T	41 (7.45)	7 (5.47)	0.67 (0.37–1.23)	0.19	28 (8.38)	1.18 (0.83–1.68)	0.36
C	509 (92.55)	121 (94.53)			306 (91.62)		
TT	2 (0.36)	0 (0)	0.81 (0.04–16.98)	0.48	2 (0.60)	1.69 (0.24–12.09)	0.59
TC	77 (14.0)	13 (10.16)	0.69 (0.37–1.28)	0.23	53 (15.87)	1.17 (0.80–1.71)	0.43
CC	471 (85.64)	115 (89.84)	1 (Reference)		279 (83.53)	1 (Reference)	
*rs641738* (*MBOAT7*)							
T	238 (43.27)	53 (41.41)	0.94 (0.71–1.24)	0.65	149 (44.61)	1.05 (0.87–1.28)	0.61
C	312 (56.73)	75 (58.59)			185 (55.39)		
TT	108 (19.64)	20 (15.63)	0.79 (0.45–1.43)	0.44	69 (20.66)	1.10 (0.75–1.62)	0.62
TC	261 (47.45)	66 (51.56)	1.11 (0.72–1.70)	0.65	160 (47.90)	1.05 (0.77–1.43)	0.76
CC	181 (32.91)	42 (32.81)	1 (Reference)		105 (31.44)	1 (Reference)	

OR—adjusted odds ratio; CI—confidence interval; SNP—single nucleotide polymorphism.

**Table 3:** Distribution of the *TMS6SF2* and *MBOAT7* SNPs alleles and genotypes in alcohol and HCV induced liver cirrhosis groups.

Allele/Genotype	Controls (*n* = 550)	Alcoholic Cirrhosis (*n* = 171)			HCV Induced Cirrhosis (*n* = 120)		
	*n* (%)	*n* (%)	aOR (95% CI)	*p*	*n* (%)	aOR (95% CI)	*p*
*rs58542926* (*TM6SF2*)							
T	41 (7.45)	14 (8.09)	1.13 (0.72–1.77)	0.60	9 (7.50)	0.96 (0.56–1.65)	0.88
C	509 (92.55)	157 (91.81)			111 (92.50)		
TT	2 (0.36)	0 (0)	0.66 (0.03–13.86)	0.44	1 (0.83)	2.26 (0.20–25.21)	0.49
TC	77 (14.0)	28 (16.37)	1.21 (0.75–1.93)	0.44	15 (12.50)	0.88 (0.49–1.60)	0.68
CC	471 (85.64)	143 (83.63)	1 (Reference)		104 (86.67)	1 (Reference)	
*rs641738* (*MBOAT7*)							
T	238 (43.27)	72 (42.11)	0.96 (0.75–1.23)	0.74	55 (45.83)	1.11 (0.83–1.46)	0.48
C	312 (56.73)	99 (57.89)			65 (54.17)		
TT	108 (19.64)	31 (18.13)	0.91 (0.55–1.50)	0.72	23 (19.17)	1.17 (0.65–2.09)	0.60
TC	261 (47.45)	83 (48.54)	0.99 (0.68–1.47)	0.99	64 (53.33)	1.35 (0.85–2.13)	0.21
CC	181 (32.91)	57 (33.33)	1 (Reference)		33 (27.50)	1 (Reference)	

OR—adjusted odds ratio; CI—confidence interval; SNP—single nucleotide polymorphism; HCV—hepatitis C virus.

**Table 4:** Combined analysis of *PNPLA3* and *TM6SF2* genotypes.

**Liver Fibrosis**					
***rs738409* (*PNPLA3*)**	***rs58542926*** **(*TM6SF2*)**	**Cases (%)**	**Controls (%)**	**aOR (95% CI)**	***p***
CC	CC	65 (87.83)	241 (87.31)	1.05 (0.48–2.29)	0.16
CC	TC+TT	9 (12.16)	35 (12.68)		
GC+GG	CC	50 (92.59)	103 (82.4)	2.66 (0.87–8.16)	0.04
GC+GG	TC+TT	4 (7.4)	22 (17.6)		
**Liver Cirrhosis**					
***rs738409* (*PNPLA3*)**	***rs58542926*** **(*TM6SF2*)**	**Cases (%)**	**Controls (%)**	**aOR (95% CI)**	***p***
CC	CC	121 (85.82)	241 (87.32)	0.88 (0.49–1.59)	0.67
CC	TC+TT	20 (14.18)	35 (12.68)		
GC+GG	CC	107 (83.59)	103 (82.40)	1.09 (0.57–2.10)	0.80
GC+GG	TC+TT	21 (16.41)	22 (17.60)		
**Alcoholic Cirrhosis**					
***rs738409* (*PNPLA3*)**	***rs58542926*** **(*TM6SF2*)**	**Cases (%)**	**Controls (%)**	**aOR (95% CI)**	***p***
CC	CC	63 (86.30)	241 (87.32)	0.92 (0.43–1.95)	0.82
CC	TC+TT	10 (13.70)	35 (12.68)		
GC+GG	CC	57 (80.28)	103 (82.40)	0.87 (0.41–1.83)	0.71
GC+GG	TC+TT	14 (19.72)	22 (17.60)		
**HCV Induced Cirrhosis**					
***rs738409* (*PNPLA3*)**	***rs58542926*** **(*TM6SF2*)**	**Cases (%)**	**Controls (%)**	**aOR (95% CI)**	***p***
CC	CC	44 (93.62)	241 (87.32)	2.13 (0.63–7.23)	0.10
CC	TC+TT	3 (6.38)	35 (12.68)		
GC+GG	CC	36 (83.72)	103 (82.40)	1.10 (0.43–2.79)	0.18
GC+GG	TC+TT	7 (16.28)	22 (17.60)		

OR—adjusted odds ratio; CI—confidence interval.

**Table 5:** Combined analysis of *PNPLA3* and *MBOAT7* genotypes.

**Fibrosis**					
***rs738409 (PNPLA3)***	***rs641738 (MBOAT7)***	**Cases (%)**	**Controls (%)**	**aOR (95% CI)**	***p***
CC	CC	23 (31.51)	87 (31.52)	0.99 (0.57–1.741)	1.00
CC	TC+TT	50 (68.49)	189 (68.48)		
GC+GG	CC	18 (32.73)	43 (34.40)	0.93 (0.47–1.82)	0.83
GC+GG	TC+TT	37 (67.27)	82 (65.60)		
**Cirrhosis**					
***rs738409 (PNPLA3)***	***rs641738 (MBOAT7)***	**Cases (%)**	**Controls (%)**	**aOR (95% CI)**	***p***
CC	CC	57 (40.43)	87 (31.52)	1.47 (0.97–2.25)	0.07
CC	TC+TT	84 (59.57)	189 (68.48)		
GC+GG	CC	32 (25.00)	43 (34.40)	0.64 (0.37–1.09)	0.10
GC+GG	TC+TT	96 (75.00)	82 (65.60)		
**Alcoholic Cirrhosis**					
***rs738409 (PNPLA3)***	***rs641738 (MBOAT7)***	**Cases (%)**	**Controls (%)**	**aOR (95% CI)**	***p***
CC	CC	31 (42.47)	87 (31.52)	1.60 (0.95–2.72)	0.08
CC	TC+TT	42 (57.53)	189 (68.48)		
GC+GG	CC	18 (25.35)	43 (34.40)	0.65 (0.34–1.96)	0.19
GC+GG	TC+TT	53 (74.65)	82 (65.60)		
**HCV Induced Cirrhosis**					
***rs738409 (PNPLA3)***	***rs641738 (MBOAT7)***	**Cases (%)**	**Controls (%)**	**aOR (95% CI)**	***p***
CC	CC	18 (38.30)	87 (31.52)	1.35 (0.71–2.56)	0.36
CC	TC+TT	29 (61.70)	189 (68.48)		
GC+GG	CC	8 (18.60)	43 (34.40)	0.44 (0.19–1.02)	0.02
GC+GG	TC+TT	35 (81.40)	82 (65.60)		

OR—adjusted odds ratio; CI—confidence interval.
